# The effect of breakfast cereal consumption on adolescents' cognitive performance and mood

**DOI:** 10.3389/fnhum.2013.00789

**Published:** 2013-11-20

**Authors:** Margaret A. Defeyter, Riccardo Russo

**Affiliations:** ^1^Department of Psychology, Northumbria UniversityNewcastle upon Tyne, UK; ^2^Department of Psychology, University of EssexEssex, UK

**Keywords:** adolescent, cognitive performance, breakfast, mood, cognitive load

## Abstract

The aim of the current study was to investigate the effect of breakfast consumption on cognitive performance and mood in adolescents, and any interaction that breakfast consumption might have with cognitive load. The rationale for this approach was that the beneficial effects of any intervention with regard to cognitive function may be more readily apparent when more demands are placed on the system. Furthermore, as skipping breakfast is particularly prevalent within this age group, thus, we focused on adolescents who habitually skip breakfast. Cognitive load was modulated by varying the level of difficulty of a series of cognitive tasks tapping memory, attention, and executive functions. Mood measured with Bond–Lader scales (1974) as well as measures of thirst, hunger, and satiety were recorded at each test session both at baseline and after the completion of each test battery. Forty adolescents (mean age = 14:2) participated in this within-subjects design study. According to treatment, all participants were tested before and after the intake of a low Glycaemic index breakfast (i.e., a 35 g portion of AllBran and 125 ml semi-skimmed milk) and before and after no breakfast consumption. Assessment time had two levels: 8.00 am (baseline) and 10.45 am. The orders of cognitive load tasks were counterbalanced. Overall it appeared that following breakfast participants felt more alert, satiated, and content. Following breakfast consumption, there was evidence for improved cognitive performance across the school morning compared to breakfast omission in some tasks (e.g., Hard Word Recall, Serial 3's and Serial 7's). However, whilst participants performance on the hard version of each cognitive task was significantly poorer compared to the corresponding easy version, there was limited evidence to support the hypothesis that the effect of breakfast was greater in the more demanding versions of the tasks.

## Introduction

The importance of breakfast consumption in terms of nutritional benefits has been well documented (Smith et al., 1999). Conversely, skipping breakfast has been associated with increased levels of snack food consumption (Billion et al., 2002), and increased likelihood of being overweight or obese (Dubois et al., 2006; Timlin et al., 2008; Croezen et al., 2009). Skipping breakfast during adolescence has also been associated with unhealthy lifestyles such as alcohol, tobacco, and substance use (Revicki et al., 1991; Isralowitz and Trostler, 1996).

In addition to physical health and nutritional benefits, cognitive scientists have investigated the effects of breakfast consumption on cognitive function and the specific cognitive processes that are affected. The majority of these studies have examined the effects of breakfast skipping in adults and children (typically 8–11 year olds).

Several experimental studies have suggested that, in both adults and children, behavior and cognitive performance is improved after consumption of breakfast compared to omission of breakfast. For example, research has shown that breakfast is associated with short-term improvements to memory (Smith et al., 1994; Vaisman et al., 1996; Benton and Parker, 1998; Wesnes et al., 2003); long-term memory (Chandler et al., 1995); attention (Wesnes et al., 2003; Ingwersen et al., 2007); mood (Smith et al., 1999; though see Benton et al., 2001); arithmetic (Powell et al., 1998); creativity (Wyon et al., 1997); and behavior (Bro et al., 1994). Despite this wealth of evidence supporting a link between breakfast consumption and cognitive benefit some studies have reported no benefit of breakfast consumption over breakfast omission (e.g., Dickie and Bender, 1982; Cromer et al., 1990; Lopez et al., 1993; for a review see Rampersaud et al., 2005) and some studies have produced rather mixed results (e.g., Smith et al., 1994). Furthermore, in general, the data are less supportive for the effects of breakfast on cognitive functions such as attention, problem-solving, and reading compared to memory (Rampersaud et al., 2005). Also, there appears to be no consensus on the specific cognitive processes that are affected by breakfast consumption (e.g., Dye et al., 2000).

A number of nutritional mechanisms have been proposed in order to explain the effects of breakfast consumption and composition on cognitive function. For example, Widenhorn-Müller et al. (2008) suggested that alleviating hunger improves mood and subsequently cognitive performance. Other authors have focused on the key role of glucose as a mediator for cognitive function, primarily as glucose is the only fuel that can be used directly by the brain. Whilst breakfast consumption/omission may not have a significant effect on low cognitive loads tasks, involving mostly information processing; high cognitive load tasks require an increase in the metabolic resources to successfully complete the task (Cooper et al., 2011). Hence the beneficial effects of breakfast consumption compared to breakfast omission may be elucidated under condition of high cognitive load. However, many of the studies, cited above, have used cognitive batteries in which cognitive load has not been investigated. Hence, conflicting findings may, in part, result from studies employing tasks of varying levels of difficulty. Previous studies have also varied in terms of research design and cognitive measures; breakfasts served; timing of post-consumption tests; and socio-economic status and the age of the participants.

Although numerous studies have examined the effect of breakfast vs. breakfast omission in adults and middle childhood there is a paucity of studies conducted with adolescent populations. There are a number of reasons to look specifically at the adolescent populations, and the four main reasons (rapid period of growth, complexity of academic work, tendency to skip breakfast, and ratio of brain size to body weight) are further discussed in Cooper et al. (2011). We conducted a review of the literature and found only two papers that directly examined breakfast consumption vs. breakfast omission in adolescents (Widenhorn-Müller et al., 2008; Cooper et al., 2011); although other studies have manipulated glycaemic index of breakfast (e.g., Smith and Foster, 2008). Widenhorn-Müller et al. (2008) employed a crossover design involving 104 pupils between 13 and 20 years of age. Pupils were tested both after a standardized breakfast consisting of 60 g of whole wheat bread, 20 g of butter, 20 g of nougat spread, and 30 g of strawberry jam and tested without breakfast. Water and unsweetened peppermint tea were offered “*ad libitum*.” Pupils completed pen and pencil standardized tests of attention and concentration, and tests of verbal and spatial memory. Mood measures were assessed by a self-administered questionnaire. The results showed breakfast had a significant effect on self-reported alertness and male pupils reported feeling more positive. Breakfast also had a significant effect on accuracy scores on a test of visuospatial memory in males, but no effect on sustained attention or verbal memory. However, due to the cognitive tests being conducted using pen and paper, only the accuracy scores of the cognitive tests were reported so it is not possible to tell whether breakfast had an effect on the speed of responses or whether there was any speed-accuracy trade-off. Moreover, the authors did not control for habitual breakfast consumption. This may be an important confounding variable in studies that pit a standard breakfast condition against a no breakfast condition. Furthermore, cognitive measures were made immediately post-breakfast, thus, potentially masking the beneficial effects of breakfast that are not apparent until later in the morning (Hoyland et al., 2009).

Cooper et al. (2011) also examined the effects of breakfast consumption vs. breakfast omission on adolescent's cognitive function and mood. Their study used a randomized crossover design with trials scheduled 7 days apart. Participants were provided with a range of breakfast foods from which they could choose “*ad libitum*.” Participants were asked to complete a mood questionnaire and a range of cognitive tasks; including a visual search task, the Stroop test, and the Sternberg paradigm. Each cognitive task had two levels of difficulty, apart from the Sternberg paradigm that had three levels. Results showed that accuracy on the more complex level of the visual search task was higher following breakfast consumption compared to breakfast omission. Across the morning, participants showed better performance on the Stroop test and responses on the Sternberg paradigm following breakfast consumption compared to breakfast omission. Breakfast consumption also had a beneficial effect on a number of the self-report measures.

Overall, across studies there is emerging evidence that breakfast is beneficial in terms of self-report measures; although the effects of breakfast consumption on cognitive function in adolescents appear to be rather mixed. One possibility is that differences in the findings relating to cognitive function are a result of the different breakfasts provided. For example, Cooper et al. (2011) used an “*ad libitum*” breakfast to allow participants to consume a breakfast similar to their habitual breakfast intake, whilst Widenhorn-Müller et al. (2008) used a standardized breakfast popular in Germany. For the current study it was decided to serve a low Glycaemic Index (GI) breakfast for two main reasons. First, given that little is known about the effect of breakfast composition on adolescents' cognitive function, providing a standardized low GI breakfast would allow the researchers to exert exact control over participants' nutritional intake at breakfast. Second, consumption of a low GI breakfast, compared to a high GI breakfast, has been shown to benefit cognitive function in both adults (Benton et al., 2003), children (Mahoney et al., 2005; Ingwersen et al., 2007), and adolescents (Cooper et al., 2012).

Unlike the aforementioned studies, the present study focused on adolescents who habitually skip breakfast as it has been found that skipping breakfast is particularly prevalent within this age group (Videon and Manning, 2003). Thus, rather than having a sample comprised of adolescents who habitually consume breakfast, the current study sampled only from adolescents who routinely skip breakfast. The skipping of breakfast within this age group has often been attributed to the switch to independent, hurried lifestyle and the reliance on fast-food sources of food and the consumption of snacks (Larson et al., 2009). In addition to the nutritional impact of this lifestyle, young adults are often under high levels of stress as a result of lifestyle changes (Croft et al., 1986). Furthermore, there have been relatively few studies that have examined the effect of breakfast in adolescents [for a review see Hoyland et al. (2009)], and none that have specifically focused on breakfast skippers.

The aim of the current study was to investigate the effect of breakfast consumption on cognitive performance and mood and any interaction that breakfast consumption might have with differing levels of cognitive load in 13–15 years old using a randomized crossover design. The underlying rationale for this approach was that the beneficial effects of any intervention with regard to cognitive function may be more readily apparent when more demands are placed on the system. Following the recommendation of Schmitt et al. (2005) and Westenhoefer et al. (2004) this study employed a cognitive test battery that encompassed a range of cognitive tests that have been shown to be sensitive to dietary manipulation. Cognitive load was modulated by manipulating task difficulty. In this instance lower vs. higher cognitive load was included as a within subjects factor in the design, with half of the participants undertaking the less demanding version of all of the tasks first and the other half the more demanding versions of the tasks first.

## Methods

### Participants

Forty adolescents (mean age = 14:2, range 13:2–15:6 years; 21 females and 19 males) whom did not habitually consume breakfast participated. We intentionally sampled from a small age range as neuroanatomical studies have shown that young, middle, and late adolescents differ in brain maturation (Giedd, 2008), and vary in glucose metabolism (Chugani, 1998). Participants were recruited from students studying at an inner-city high school in the North East of England. The school served predominantly lower-middle class children. We further controlled for socioeconomic status by looking at the Level of Parental Education (LPE) (Lien, 2006). LPE can be used as a reliable estimate of socio economic status (Hauser, 2008) and is associated to educational performance and attainment, and breakfast behaviors. We specifically targeted adolescents of lower-socio economic status as research has suggested that these participants are more likely to skip breakfast (e.g., Affenito et al., 2005; Utter et al., 2007); suggesting that future interventions may need to specifically target people in this demographic. In order to be included in the current study all adolescents had to have parents that had not undertaken any higher educational studies. Habitual breakfast consumption was measured by asking adolescents to complete online food diaries across 5 school days prior to commencing the test phase of the study. Although, there is no clear universal definition of breakfast (e.g., Rampersaud et al., 2005), qualitative research suggests that adolescents have a well-defined idea of the types of food that constitute breakfast as well as the time breakfast is consumed (Chapman et al., 1998; Mullan and Singh, 2010). For the purpose of the present study, breakfast was defined as any food consumed between waking and school lunchtime. Only young people who met the above criteria and had skipped breakfast on 5 consecutive school days in the week prior to commencement of testing were invited to participate in the main test phase of the study. Prior to testing all pupils completed a health screen questionnaire. All participants were reported to be healthy and BMI [calculated by dividing body mass (kg) by the square of the height (m^2^)] was used to recruit a sample that fell within the normal BMI (Cole et al., 2000) (see Table 1). All participants were free from any food allergy or use of prescription drugs, and all participants spoke English as a first language and no participants had any special educational needs.

**Table 1:** **Anthropometric characteristics of participants**.

	***n***	**Age (years)**	**Height (cm)**	**Body mass (Kg)**	**BMI (kg/m^2^)**	**Waist circumference**
Male	19	14.1 ± 0.5	169.1 ± 8.3	60.3 ± 2.3	21.3 ± 2.4	74.7 ± 6.0
Female	21	14.3 ± 0.5	162.5 ± 5.1	56.8 ± 6.2	21.6 ± 2.8	66.5 ± 6.4
Total	40	14.2 ± 0.5	165.2 ± 6.4	58.6 ± 4.7	21.4 ± 2.6	69.6 ± 6.1

All values are mean ± standard deviation.

### Design

The study was approved the Life Sciences Ethics Committee at Northumbria University. Participants were recruited through one school and in accordance with the British Psychological Society Code of Ethics. Written consent was obtained from the head teacher, parents or guardians, and pupils. The short-term effects of cereal consumption on cognition were investigated using a crossover design in which 40 adolescents were given a ready-to-eat breakfast cereal or no breakfast cereal. According to treatment, all participants were provided with 35 g of Allbran (low GI breakfast cereal selected from an international table of glycaemic index; Foster-Powell et al., 2002) and 125 ml of skimmed milk or no breakfast. Adolescents were tested prior to consumption of breakfast (baseline) and then 120 min post start of breakfast consumption. The order of breakfast consumption and breakfast omission was fully counterbalanced, so that half of the children consumed breakfast on the first test day and omitted breakfast on the second test day while the remaining children were presented with the same conditions but in the reverse order. Half of the participants completed the low cognitive load tests followed by the high cognitive load tests, and the remaining participants completed the high cognitive load tests followed by the low cognitive load tests, thus, participants acted as their own controls.

A sample size of 40 was selected to obtain a statistical power of 0.80 on the assumption of a small to medium size, i.e., partial-η^2^ = 0.05, of the effect of the primary variables of interest (i.e., the differential effect of breakfast, and the breakfast by task load interaction, between baseline and 120 min post start of breakfast consumption) and of a correlation *r* = 0.5 between repeated measures.

### Measures

The test battery comprised a series of computerized tasks derived from standard psychometric measures. All tasks were programmed in JAVA language and the timing of the test battery and reaction times were made independently of the computer's internal timing. The presentations of high and low cognitive load tasks were counterbalanced across participants. The tasks utilized in the current study comprised: Delayed Word recall; Choice reaction time; Rapid Visual Information Processing; Stroop; and Serial subtractions. In addition to the test battery, participants were asked to complete the Bond–Lader mood scale, and visual analog scales for thirst, hunger, and satiety.

#### Delayed word recall

Participants were presented with lists of 15 words taken from Snodgrass and Vanderwart (1980). Words were matched for familiarity and word length. Stimulus duration was one second, as was the inter-stimulus duration. The cognitive load of this task was modulated by presenting words that can be categorized (low cognitive load) and words that cannot be categorized (high cognitive load). At the end of the entire test battery participants were asked to write down as many words as they remembered from both of the original lists and these items were scored according to whether they appeared on the “categorized” or “uncategorized” list.

#### Choice reaction time

Choice reaction time tasks is a widely used test of attention and has previously demonstrated sensitivity to the improvements and decrements seen in cognitive performance following a number of food components and dietary supplements. Fifty stimuli were presented with an inter-stimulus interval that varied randomly between 1 and 3.5 s. Accuracy and reaction times (ms) were recorded. The cognitive load of this task was modulated by presenting either two choices of response (low load) or four choices of response (high load). In the low cognitive load version of this task participants were required to press the “x” key on a computer keyboard as soon as they saw the letter “N” and the “?” key each time they saw the letter “M.” In the high load version of this task participants were required to also press the “c” key as soon as they saw the letter “B” and the “>” key as soon as they saw the letter “V” presented on a computer screen.

#### Rapid visual information processing task (RVIP)

Participants were instructed to monitor a continuous series of digits for targets of three consecutive odd or three consecutive even digits. The participant responded to the detection of a target string by pressing a response key as quickly as possible. The task was continuous and lasted for 5 min, with 8 correct target strings being presented in each minute. Dependent variables include the number of target strings correctly detected (hits), number of false alarms, and reaction time for hits. The cognitive load of this task was modulated by altering the rate at which the digits were presented: 80 per min (low load) or 100 per min (high load).

#### Stroop color-word test (Stroop, 1935)

Participants were presented with words describing one of four colors (“RED,” “YELLOW,” “GREEN,” “BLUE”) on a computer screen. The cognitive load of this task was modulated by presenting either congruent (low load) or incongruent stimuli (high load). The participant was instructed to press the corresponding button as quickly as possible in order to identify the font color (e.g., if the word “Red” is presented in a blue font, the correct response would be to press the “blue” button).

#### Serial subtractions

A modified, 2 min, computerized version of the serial subtraction tests was utilized. In this task, participants were asked to count backwards in threes or sevens from the given randomly generated number, as quickly and accurately as possible, using the numeric keypad to enter each response. Participants were also instructed verbally that if they make a mistake they should carry on subtracting from the new incorrect number. Each three-digit response was entered via the numeric keypad with each digit being represented on screen by an asterisk. Pressing the “enter” key signals the end of each response and clears the three asterisks from the screen. The task was scored for total number of subtractions and number of errors. In the case of incorrect responses, subsequent responses were scored as positive if they were correct in relation to the new number. The cognitive load of this task was modulated by instructing participants to either subtract “threes” (low load) or “sevens” (high load).

#### Mood (Bond and Lader, 1974)

Mood was assessed with Bond–Lader scales following completion of the cognitive test battery. Scores from the 16 Bond–Lader visual analog scales were combined as recommended by the authors to form three mood factors: “alert,” “calm” and “contentment.” The scales were completed by participants placing a cross with the mouse and cursor on a 100 mm line displayed on a computer screen between the description “not at all” and “extremely” for each of the listed mood states (i.e., alert, content, and calm). Each mood factor was scored as a percentage along the line demoting more of the relevant adjective.

#### Visual analog scales

Hunger, Thirst and Satiety were assessed using visual analog scales (1–100; with 1 indicating the lowest levels). As in the Bond–Lader Mood Scales, participants completed the VAS by placing a cross with the mouse and cursor on a 100 mm line displayed on a computer screen. VAS were scored as percentage along the line denoting more of the relevant adjective.

## Procedure

All participants were tested in a quiet room within their High School. The researcher visited the school on three separate occasions and participants were tested individually on laptops. Each participant undertook a familiarization session which preceded the start of the main test phase of the study by 1 week. The purpose of each cognitive test was explained to participants and a demonstration given. Participants then completed the full battery of cognitive tests which lasted about 30 min. Throughout the familiarization phase, researchers were available to answer any questions. Participants also completed the Bond–Lader Mood Scales and VAS measuring hunger, thirst, and satiety. This enabled participants to become familiar with the test protocol. In this visit the researcher obtained participant's informed consent; and collected parental consent forms. Participants were also given a health screening questionnaire to be completed and signed both by the participant and their parents/guardian. Participant's height and body mass were also measured. These measures allowed the determination of BMI. In order to ensure confidentiality participants were provided with a stamped addressed envelope in which to return the questionnaire directly to the research team. Demographic data were collected and participants were randomly allocated to treatment conditions. Participants were reminded that, for the testing session, they would need to arrive at their school for 8.00 am, having consumed no caffeine for at least 12 h previously and no food from midnight.

**Figure 1:**
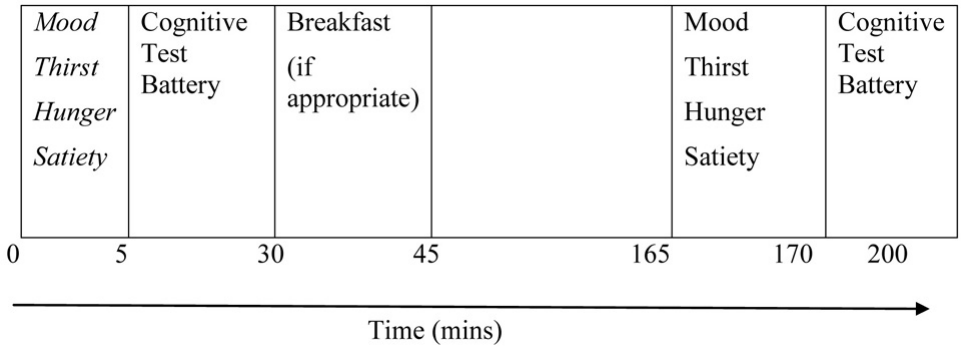
**Experimental Protocol**.

Following standard protocol, the two testing visits took place 1 week apart (Widenhorn-Müller et al., 2008), and participants reported to school at 8 am following an overnight fast from midnight the evening before the trial. Upon arrival at school participants were asked to complete a computerized cognitive test battery and the mood questionnaire and rate satiety, thirst, and hunger. Each cognitive test was preceded by 6 practice stimuli in order to re-familiarize participants with each individual cognitive test. Participants were then provided with a breakfast (breakfast trial) at 8.30 am or no breakfast (no breakfast trial). No additional help or feedback was provided on any of the test trials. They were given 15 min to consume breakfast or 15 min of resting in the no breakfast trial. Following testing, participants started their normal lessons and then returned for testing again at 10.45 am. Participants in both groups were allowed to drink water across the school morning, if desired. The experimental protocol is shown in Figure 1.

### Data analysis

Primary analyses consisted of repeated-measures analysis of variance (ANOVA) with two within-subjects variables: breakfast trial (breakfast vs. no breakfast) and time (pre- vs. post-breakfast consumption). This type of analysis was applied to each dependent variable used in the study. Moreover, in the case of cognitive tasks, this analysis was applied first to the low cognitive load and then to the high cognitive load condition. Further analyses to assess any differential effect of the cognitive load variable were conducted only if there was at least a significant breakfast by time interaction either at the low or at the high level of load version of the task being considered. A significance level of 0.05 was used through the study and effect sizes (*partial*-η^2^—indicated in the text simply as η^2^) were reported for *F* ratios larger than one. As a rule of thumb *partial*-η^2^ of the following magnitudes: 0.01, 0.06, 0.14, correspond to small, medium, and large effect sizes, respectively. For each ANOVA, the outcome of the interaction between breakfast trial by time is reported first, followed by the main effects.

For each measure of cognitive function, mood, thirst, hunger and satiety, preliminary analyses had been conducted to ascertain whether there was any significant effect of either gender or trial order. Since these factors did not have any significant effect, data had been collapsed across gender and trial order in all subsequent analysis. Factorial repeated within-subjects and mixed models ANOVAs were conducted using SPSS version 18.

Further preliminary analyses were conducted to test if there was any significant difference at baseline (i.e., the first measurement of the morning) between breakfast and no breakfast conditions for each dependent variable used. The only significant difference emerged in the Serial 3's task. However, given the large amount of pair-wise comparisons being performed this significant difference may simply reflect a Type 1 statistical error.

## Results

### Self report measures

#### Alertness

Analysis revealed a significant and a rather large effect of the breakfast by time interaction [*F*_(1, 39)_ = 12.89, *p* < 0.05, η^2^ = 0.249] with alertness increasing following breakfast consumption (41.08 vs. 50.12) compared to a decrease in alertness in the no breakfast trials [40.37 vs. 36.44 (see also Table 2 for this and the other self report measures)]. There was also a significant main effect of breakfast [*F*_(1, 39)_ = 11.32, *p* < 0.05, η^2^ = 0.225] with participants reporting feeling more alert on the breakfast trial compared to the no breakfast trial (45.60 vs. 38.40, respectively). There was no significant main effect of time [*F*_(1, 39)_ = 2.13, *p* > 0.05, η^2^ = 0.05].

**Table 2:** **Performance on Mood dimensions, Hunger, Satiety, Thirst and on Cognitive Tasks as a factor of Breakfast and Test Time**.

**Task**	**Breakfast**	**Breakfast**	**No breakfast**	**No breakfast**
	**Time 1**	**Time 2**	**Time 1**	**Time 2**
Alertness	41.08 ± 12.48	50.12 ± 12.48	40.37 ± 15.42	36.44 ± 14.38
Calm	64.21 ± 12.28	63.12 ± 12.86	63.10 ± 14.58	55.16 ± 8.72
Contentment	55.40 ± 13.85	61.60 ± 9.14	53.90 ± 14.37	51.44 ± 10.56
Hunger	66.02 ± 11.83	49.18 ± 13.78	61.30 ± 16.00	56.35 ± 21.11
Satiety	28.37 ± 13.92	48.10 ± 14.51	31.93 ± 15.48	38.03 ± 19.74
Thirst	58.15 ± 17.85	50.27 ± 12.03	55.13 ± 16.61	54.60 ± 17.07
Easy word recall (% correct)	66.00 ± 14.46	64.67 ± 12.09	60.50 ± 17.56	63.17 ± 11.09
Hard word recall (% correct)	50.33 ± 14.34	54.08 ± 13.51	51.75 ± 15.36	44.00 ± 12.32
Easy 2 choice (% correct)	97.15 ± 3.35	96.45 ± 2.92	96.65 ± 3.18	96.70 ± 2.62
Easy 2 choice reaction time (ms)	427.78 ± 63.76	422.47 ± 63.76	421.43 ± 59.86	423.01 ± 61.27
Hard 4 choice	98.08 ± 2.92	98.13 ± 2.56	98.75 ± 2.04	98.38 ± 2.13
Hard 4 choice reaction time (ms)	475.75 ± 61.76	466.26 ± 60.85	481.64 ± 60.70	477.52 ± 84.31
Easy stroop (% correct)	98.12 ± 2.44	97.62 ± 2.44	97.51 ± 2.65	97.73 ± 2.60
Easy stroop reaction time (ms)	857.73 ± 91.89	822.90 ± 97.05	872.63 ± 124.46	833.05 ± 152.57
Hard stroop (% correct)	98.86 ± 2.38	99.24 ± 1.24	97.14 ± 10.35	98.68 ± 2.35
Hard stroop reaction time (ms)	904.90 ± 117.04	872.05 ± 111.79	915.66 ± 228.49	869.05 ± 161.24
Easy RVIP (% correct)	58.69 ± 18.48	55.65 ± 21.88	58.55 ± 21.84	55.37 ± 23.02
Easy RVIP reaction time (ms)	506.08 ± 37.87	494.61 ± 42.31	505.99 ± 45.79	491.34 ± 41.29
Hard RVIP (% correct)	45.99 ± 15.41	49.13 ± 17.84	49.17 ± 12.99	46.50 ± 17.56
Hard RVIP reaction time (ms)	502.13 ± 38.62	502.57 ± 44.09	500.59 ± 37.08	493.15 ± 45.05
Easy serial 3's	31.13 ± 12.28	32.30 ± 13.03	35.35 ± 12.51	31.85 ± 11.47
Hard serial 7's	20.58 ± 9.69	21.80 ± 9.26	21.60 ± 8.55	19.20 ± 8.65

#### Calm

The analysis on self-report measure of calmness showed a significant breakfast by time interaction [*F*_(1, 39)_ = 5.96, *p* < 0.05, η^2^ = 0.133]. All participants reported feeling less calm across time, although this effect was far more pronounced in the no breakfast trials (63.10 vs. 55.16) compared to the breakfast trials (64.21 vs. 63.12).

There were also significant main effect of breakfast [*F*_(1, 39)_ = 11.21, *p* < 0.05, η^2^ = 0.223; 63.7 vs. 59.1 for breakfast and no breakfast conditions, respectively]; and a significant main effect of time [*F*_(1, 39)_ = 6.96, *p* < 0.05, η^2^ = 0.152; 63.7 vs. 59.1 for pre and post breakfast conditions, respectively].

#### Contentment

The analysis revealed a significant and a rather large effect of the interaction between breakfast and time [*F*_(1, 39)_ = 9.53, *p* < 0.05, η^2^ = 0.196], with participants in the breakfast trials reporting a greater level of contentment later in the morning (55.4 vs. 61.6), whilst participants in the no breakfast trials reported lower level of contentment later in the morning (53.9 vs. 51.4).

There was no significant main effect of time [*F*_(1, 39)_ = 1.54, *p* > 0.05, η^2^ = 0.038], but there was a significant main effect of breakfast [*F*_(1, 39)_ = 14.34, *p* < 0.05, η^2^ = 0.269; 58.5 vs. 52.7 for breakfast and no breakfast conditions, respectively].

#### Hunger

There was a significant breakfast by time interaction [*F*_(1, 39)_ = 6.73, *p* < 0.05, η^2^ = 0.147], with participants in the breakfast trials showing a larger reduction in self-reported hunger across the morning (66.0 vs. 49.2) compared to participants in the no breakfast trials (61.3 vs. 56.4). Moreover, there was a significant and rather large effect of time [*F*_(1, 39)_ = 23.45, *p* < 0.05, η^2^ = 0.375; 63.7 vs. 52.8 for pre and post breakfast conditions, respectively), while the main effect of breakfast was not significant (*F* < 1).

#### Satiety

Analysis showed that there was a significant and large effect of the breakfast by time interaction [*F*_(1, 39)_ = 11.06, *p* < 0.05, η^2^ = 0.221] with participants in the breakfast trials reporting feeling fuller across the school morning (28.4 vs. 48.1) than participants in the no breakfast trials (31.9 vs. 38.0). There was a significant and very large effect of time [*F*_(1, 39)_ = 36.21, *p* < 0.05, η^2^ = 0.481; 30.2 vs. 43.1, for pre and post breakfast conditions, respectively], but no significant effect of breakfast [*F*_(1, 39)_ = 2.89, *p* > 0.05, η^2^ = 0.069].

#### Thirst

There was no significant breakfast by time interaction [*F*_(1, 39)_ = 2.81, *p* > 0.05, η^2^ = 0.067], with reported thirst in the breakfast trials (58.2 vs. 50.3) and in the no breakfast trials (55.1 vs. 54.6), neither significant main effects of time (*F* < 1) or of breakfast [*F*_(1, 39)_ = 3.66, *p* > 0.05, η^2^ = 0.075].

### Delayed word recall

#### Easy word recall

Participants performance on all of the cognitive tasks are reported in Table 2. There was no significant breakfast by time interaction [*F*_(1, 39)_ = 1.03, *p* > 0.05]. Participants in the breakfast trials across the school morning recalled (66.00 vs. 64.67) while in the no breakfast trials the means were (60.5 vs. 63.17). There was a significant main effect of breakfast trial [*F*_(1, 39)_ = 4.09, *p* < 0.05, η^2^ = 0.095], with significantly more correct words recalled in the breakfast trials compared to the no breakfast trials (65.33 vs. 61.83). However, there was no main effect of time (*F* < 1).

#### Hard word recall

There was a significant and substantially large effect of the breakfast by time interaction [*F*_(1, 39)_ = 13.96, *p* < 0.05, η^2^ = 0.264]. Participants recalled more correct words in the breakfast trials across the school morning (50.33 vs. 54.08) compared to the no breakfast trials under which performance decreased (51.7 vs. 44.0). There was a significant effect of breakfast [*F*_(1, 39)_ = 5.73, *p* < 0.05, η^2^ = 0.128] and no significant main effect of time (*F* < 1).

When easy and hard tasks were compared it appeared that there was, as expected, a significant and very large effect of difficulty, [*F*_(1, 39)_ = 70.83, *p* < 0.05, η^2^ = 0.645], indicating that more words were recalled in the easy (63.58) than in the hard condition (50.04). More interestingly the three-way interaction was significant and rather large in terms of the magnitude of the size of its effect, [*F*_(1, 39)_ = 12.68, *p* < 0.05, η^2^ = 0.245], indicating that differences in performance in favor of the breakfast condition emerged only when the recall task was made harder.

### Reaction time

#### Easy choice reaction time

Analysis of accuracy and reaction time data showed no significant interaction or significant main effects (*Fs* < 1).

#### Hard choice reaction time

Analysis of accuracy and reaction time data showed no significant interaction or significant main effects (*Fs* < 1.83, *ps* > 0.05, largest η^2^ < 0.045).

### Stroop task

#### Easy stroop

In looking at accuracy data no significant interaction or significant main effects emerged (*Fs* < 1.95, *ps* > 0.05, largest η^2^ < 0.048). The analysis of the reaction time data showed that neither the interaction nor the main effect of breakfast were significant (*Fs* < 1). However, there was a significant main effect of time [*F*_(1, 39)_ = 5.99, *p* < 0.05, η^2^ = 0.133] indicating that faster reaction times occurred in the second administration of the test.

#### Hard stroop

There were no significant results on accuracy data (*Fs* < 1.75, *ps* > 0.05, largest η^2^ < 0.044), The analysis of the reaction time data showed that neither the interaction nor the main effect of breakfast were significant (*Fs* < 1). However, there was a significant main effect of time [*F*_(1, 39)_ = 4.34, *p* < 0.05, η^2^ = 0.10] indicating that faster reaction times occurred in the second administration of the test.

### RVIP

#### Easy RVIP

Analysis of accuracy and reaction time data showed no significant interaction or significant main effects (*Fs* < 3.6, *ps* > 0.05, largest η^2^ < 0.084).

#### Hard RVIP

Analysis of accuracy and reaction time data showed no significant interaction or significant main effects (*Fs* < 3.46, *ps* > 0.05, largest η^2^ < 0.081).

### Serial 3's and serial 7's

#### Serial 3's

Analysis revealed a significant interaction between breakfast and time [*F*_(1, 39)_ = 6.23, *p* < 0.05, η^2^ = 0.138], with performance decreasing across the school morning in the no breakfast trials compared to the breakfast trials. The main effects of breakfast and of time were not significant (*Fs* < 2.64, *p* > 0.05, largest η^2^ < 0.064].

#### Serial 7's

There was a significant breakfast by time interaction [*F*_(1, 39)_ = 5.25, *p* < 0.05, η^2^ = 0.119], with increased performance across the school morning in the breakfast trials and reduced performance in the no breakfast trials. None of the main effects was significant (*F* < 1).

When easy and hard tasks were compared it appeared that there was, as expected, a significant and very large effect of difficulty, [*F*_(1, 39)_ = 105.91, *p* < 0.05, η^2^ = 0.731], indicating that more correct responses were given in the serial 3's (32.66) than in the serial 7's condition (20.79). The effect of the breakfast by time interaction was significant and large in size, [*F*_(1, 39)_ = 9.17, *p* < 0.05, η^2^ = 0.19], indicating increased performance across the school morning in the breakfast trials (31.8 vs. 33.6) and reduced performance in the no breakfast trials (21.2 vs. 20.4). However, the three-way interaction was not significant, (*F* < 1), indicating that difficulty of the task did not qualify the breakfast by time interaction.

## Discussion

The number of adolescents skipping breakfast appears to be increasing (Rampersaud et al., 2005) and there is thus, a need for studies to investigate the effects of breakfast omission on cognitive function and mood. This study is important as it contributes to a limited number of studies investigating the effects of breakfast consumption in this age group. Furthermore, unlike a number of prior studies, all of the adolescents in the present study were habitual breakfast skippers from low socioeconomic backgrounds. To our knowledge this is the first study to specifically target this group. This is important, given the prevalence of adolescents who habitually skip breakfast (Dwyer et al., 2001); especially those from low socioeconomic backgrounds (Rampersaud et al., 2005).

The overall findings produced a rather mixed pattern of results. The findings of the present study clearly demonstrate that following breakfast consumption self-report measures of alertness, and contentment were higher when compared to breakfast omission. These findings replicate a number of studies that have shown breakfast consumption to have a positive effect on mood (Wesnes et al., 2003; Widenhorn-Müller et al., 2008). For example, Widenhorn-Müller et al. (2008) found an increase in alertness and information uptake following breakfast consumption compared to breakfast omission. However, unlike the present study in which alertness significantly increased both for girls and boys following breakfast consumption, Widenhorn-Müller et al., only found this effect in girls. The results of the present study are also in accordance with Wesnes et al. (2003) who also found a positive effect on self-rated alertness and contentment following breakfast consumption compared to breakfast omission in 9–16-year-olds. All participants, in the current study, reported feeling less calm across the school morning; although this effect was more pronounced following breakfast omission. These results contradict those of Cooper et al. (2011) who found no difference in self-reported calmness across breakfast conditions. Cooper and colleagues draw attention to the fact that many previous studies have used mood questionnaires specifically designed for use with adult populations and that adolescents may have difficulty in completing the scales. However, the current study successfully used a computerized version of the Bond–Lader Mood Scale and found no evidence of adolescents experiencing any difficulty in completing the scale.

As anticipated, following breakfast consumption there was a significant reduction on self-reported levels of hunger. Similarly, participants reported feeling more satiated following breakfast compared to no breakfast. There was no significant effect of breakfast consumption on thirst, but it is worthwhile remembering that participants were free to drink water across the trial period.

In looking at the findings from the cognitive function tasks the pattern of findings is not so straightforward.

### Word recall

In the easy version of this task, participants' performance in the breakfast trials outperformed participants in the breakfast omission trials. However, neither the main effect of time nor the time by breakfast interaction were significant. In the more cognitively demanding version of this task the results showed a significant time by breakfast interaction with participants recalling more correct words in the breakfast trials vs. the no breakfast trials. Moreover, when easy and hard tasks were compared more words were correctly recalled in the easy version of the task than the hard version. A three-way interaction indicated that performance in favor of the breakfast trials only emerged in the harder version of the task. These findings support the suggestion that tasks with higher cognitive demands are more sensitive to nutritional manipulations (Scholey et al., 2001; Cooper et al., 2011). Cooper et al. (2011) reported that adolescents response times, on a high load working memory test, improved 120 min post consumption of a low GI breakfast compared to breakfast omission; although there was no significant effect of breakfast on accuracy data. By contrast, adolescents in the current study showed superior accuracy, in the high cognitive load task, later in the school morning following breakfast consumption compared to breakfast omission.

### Choice reaction time

Analysis of the Choice Reaction time data found no significant main effects or interactions on either the easy or hard version of the task. These results contrast with Conners and Blouin (1983) findings that showed that adolescent's performance on attention span and vigilance improved following breakfast consumption.

### Stroop task

Analysis of the both versions of the Stroop Task data revealed no significant main effects or interactions. The results of the present study failed to replicate those of Cooper et al. (2011) who reported that while performance declined across the school morning the reduction in performance was not as noticeable following breakfast consumption. Given the role of glucose for cognitive activity (Pollitt and Matthews, 1998) and the role of the frontal lobe in determining performance in the Stroop Task it is rather surprising that we did not find an effect of breakfast consumption. A number of researchers have proposed that a high GI (high GL) breakfast results in higher blood glucose concentration and this results, in turn, in greater activation of the hypothalamic pituitary-adrenal axis, and increased frontal lobe functioning which is crucial at inhibiting the response to incongruent stimuli (e.g., Dye et al., 2000; Micha et al., 2006). However, previous studies have revealed a rather mixed pattern of findings with some studies showing that a high GI and high Glycaemic Load (GL) breakfast tends to produce better performance on this test compared to either a low GI or a low GL breakfast (e.g., Micha et al., 2008); while other reporting that GI of breakfast has no effect on adolescents performance (Micha et al., 2010); and yet another study showing that a low GI breakfast is more beneficial to adolescents performance compared to both a high GI breakfast and breakfast omission (Cooper et al., 2012).

In the current study, participants' speed of response on the Stroop test improved across time. One may argue that these findings may be due to practice effects. However, adolescents were provided with extensive training and practice during the training phase and took part in practice trials prior to each test phase, thus, reducing the likelihood of practice effects. Second, Cooper et al. (2011) also reported that adolescents' responses on the Stroop test were faster later in the morning (120 min following breakfast consumption) compared to immediately following breakfast consumption.

### RVIP

For both RVIP versions of the task there were no significant main effects and no significant interactions.

### Serial 3's and serial 7's

In both Serial 3's and Serial 7's tasks performance decreased across the school morning in the no breakfast trials compared to the breakfast trials where performance increased numerically. As anticipated there was a significant effect of task difficulty. Moreover, independently of the level of cognitive load of the task, the breakfast by time interaction was significant, indicating increased performance across the school morning in the breakfast trials and reduced performance in the no breakfast trials. However, the three-way interaction (breakfast by time by cognitive load) was not significant, indicating that difficulty of the task did not qualify the breakfast by time interaction.

### Summary and future research

Overall, the findings clearly demonstrate that task difficulty (cognitive load) mattered in general but only in some aspects of cognition. It was only in the recall task that performance appeared to be significantly modulated by the interactive combination of the effect of breakfast consumption and task difficulty; with improved performance at time two when the task was harder. Although our results partially replicate other studies in demonstrating an effect of breakfast consumption on memory (e.g., Smith et al., 1999) and attention (Wesnes et al., 2003), the findings of the current study only partially replicates those of Cooper et al. (2011) who report that breakfast consumption was particularly beneficial on more cognitively demanding tasks. Moreover, the current findings lend little support to Cooper et al.'s (2012) findings that showed improved performance in 12–14 year-olds on the Stroop test and Flanker task. Whilst the present study presents clear evidence that the hard versions of cognitive tasks were more demanding than the easy versions, the threshold at which cognitively demanding tasks become sensitive to various nutritional manipulations is currently unclear.

The findings of the current study warrant further investigation. The nutritional manipulation in the present study was the comparison of a low GI breakfast compared to no breakfast. It is possible that consumption of a low GI breakfast did not result in significantly greater glucose availability which may be required to fuel the brain during tasks of high cognitive demand (Smith and Foster, 2008). In order to address the role of glycaemic index further studies employing adolescents need to be conducted including measurements of biomarkers (e.g., blood glucose levels). In addition, unlike the study by Cooper et al. (2011) in which adolescents were provided with breakfast *ad libitum*, the current study provided a fixed amount of breakfast cereal and this may account for some of the different findings between the two studies; [although this explanation cannot account for the different findings to those of Cooper et al. (2012), where breakfast was not provided on at *ad libitum* basis].

Unlike prior studies, the current study specifically targeted adolescents from low SES who habitually skipped breakfast. Although this allowed us to focus on a specific group of participants, it also limits the generalizability of the results and as such may potentially account for differences in findings to those of Cooper et al. (2012). However, given the general rather poor health habits of this population (e.g., Benton and Nabb, 2004) it is important for researchers to examine the effects of nutritional interventions in this population.

Further studies are also required to explore the optimal timing of breakfast (see Hoyland et al., 2009). Both the current study and that of Cooper et al. (2012) assessed performance 120 min following breakfast consumption, but found quite different results. However, it should be noted that these studies tested adolescents from different SES groups and some of the cognitive tasks differed. Given the inconsistent results observed in the current literature, an important task for future studies is to carefully investigate which cognitive tasks, and associated high and low load versions of these tasks, are sensitive to nutritional manipulations in different groups. Finally, further studies need to explore why adolescents often skip breakfast in order to develop a successful intervention to tackle this unhealthy behavior.

### Conflict of interest statement

This research was funded by Kellogg's. The authors declare that the research was conducted in the absence of any commercial or financial relationships that could be construed as a potential conflict of interest.
